# Comparative Genome-Wide Transcriptional Analysis of Al-Responsive Genes Reveals Novel Al Tolerance Mechanisms in Rice

**DOI:** 10.1371/journal.pone.0048197

**Published:** 2012-10-26

**Authors:** Tomokazu Tsutsui, Naoki Yamaji, Chao Feng Huang, Ritsuko Motoyama, Yoshiaki Nagamura, Jian Feng Ma

**Affiliations:** 1 Institute of Plant Science and Resources, Okayama University, Kurashiki, Japan; 2 Genome Resource Center, Division of Genome and Biodiversity Research, National Institute of Agrobiological Sciences, Tsukuba, Ibaraki, Japan; Boston University Medical Center, United States of America

## Abstract

Rice (*Oryza sativa*) is the most aluminum (Al)-tolerant crop among small-grain cereals, but the mechanism underlying its high Al resistance is still not well understood. To understand the mechanisms underlying high Al-tolerance, we performed a comparative genome-wide transcriptional analysis by comparing expression profiling between the Al-tolerance cultivar (Koshihikari) and an Al-sensitive mutant *star1* (*SENSITIVE TO AL RHIZOTOXICITY 1*) in both the root tips and the basal roots. Exposure to 20 µM AlCl_3_ for 6 h resulted in up-regulation (higher than 3-fold) of 213 and 2015 genes including 185 common genes in the root tips of wild-type and the mutant, respectively. On the other hand, in the basal root, genes up-regulated by Al were 126 and 2419 including 76 common genes in the wild-type and the mutant, respectively. These results indicate that Al-response genes are not only restricted to the root tips, but also in the basal root region. Analysis with genes up- or down-regulated only in the wild-type reveals that there are other mechanisms for Al-tolerance except for a known transcription factor ART1-regulated one in rice. These mechanisms are related to nitrogen assimilation, secondary metabolite synthesis, cell-wall synthesis and ethylene synthesis. Although the exact roles of these putative tolerance genes remain to be examined, our data provide a platform for further work on Al-tolerance in rice.

## Introduction

Aluminum (Al) toxicity is a major factor limiting crop production on acid soils, which comprise approximately 40% of the world’s arable soils and up to 70% of potentially arable land [1]. At soil pH below 5.0, toxic forms of Al (mainly Al^3+^) are solubilized into the soil solution, which inhibit root growth and function, consequently reducing crop yields [2], [3]. However, there is a great variation for the ability to withstand Al-toxicity between plant species and cultivars within a species. To survive on acidic soils, some plant species or cultivars have evolved mechanisms to tolerate high levels of toxic Al. Many mechanisms for both Al-tolerance and -toxicity have been proposed [3].

Rice (*Oryza sativa*) is the most Al-tolerant crop among small-grain cereals [4]. A number of quantitative trait loci (QTLs) for Al-tolerance have been identified in rice by using different populations [5], but responsible QTL genes have not been isolated. Recently, through genome-wide association analysis and QTL mapping, 48 loci associated with Al^3+^ tolerance have been identified [6] in rice. On the other hand, mutant approaches have revealed an ART1-regualted Al-tolerance mechanism in rice [7]. ART1 (AL^3+^ RESISTANCE TRANSCRIPTION FACTOR 1) is a Cys2His2-type zinc-finger transcription factor [8]. ART1 is constitutively expressed in the roots and its expression is not induced by Al^3+^ treatment. ART1 regulates the expression of at least 31 genes with a cis-element [GGN(T/g/a/C)V(C/A/g)S(C/G)] (ART1-binding affinity of nucleotides with small characters is weaker than those with large characters) [9]. Among them, only six genes have been functionally characterized. *OsSTAR1* and *OsSTAR2* (*SENSITIVE TO ALUMINUM RHIZOTOXICITY 1 & 2*) encode a ATP-binding domain and a transmembrane domain, respectively, of a bacterial-type ATP binding cassette (ABC) transporter, which transports UDP-glucose [10]. The complex is implicated in cell wall modification [10]. *OsFRDL4* (*FERRIC REDUCTASE DEFECTIVE3-LIKE 4*) encodes a citrate transporter, which secretes citrate from the roots to chelate Al in the rhizosphere [11]. On the other hand, *OsNrat1* (*NRAMP ALUMINUM TRANSPORTER 1*) encodes an Al transporter localized at the plasma membrane, which transports Al into the cells [12], while *OsALS1* (*ALUMINUM SENSITIVE 1*) encodes a tonoplast-localized transporter for Al, which sequestrates Al into the vacuoles [13]. Recently, up-regulation of a Mg transporter, OsMGT1 (MAGNESIUM TRANSPORTER 1), is reported to be required for conferring Al-tolerance in rice [14]. All of these genes are specifically induced by Al and knockout of either gene results in decreased Al-tolerance, indicating their important roles in Al-tolerance. However, the mechanisms underlying high Al-tolerance in rice are not fully understood at the molecular level.

In the present study, we performed a genome-wide transcriptional analysis of Al-responsive genes in rice. By comparing transcriptional profiling between a wild-type rice and an Al-sensitive rice mutant *star1*, we found that rice possesses novel mechanisms of Al-tolerance in addition to ART1-regulated mechanism in rice.

## Materials and Methods

### Plant Materials and Growth Conditions

Seeds of wild-type rice (*Oryza sativa* cv. Koshihikari) and an Al-sensitive mutant, *star1* [10], were germinated for 2 days at 30°C. The seedlings were then transferred to a plastic net floating on a 0.5 mM CaCl_2_ solution in a 1.5 L plastic box. At day 4, the seedlings were exposed to a 0.5 mM CaCl_2_ solution (pH 4.5) containing 0 or 20 µM AlCl_3_. Root length was measured with a ruler before and after 6 h treatments. Ten seedlings were used for each treatment.

**Figure 1:**
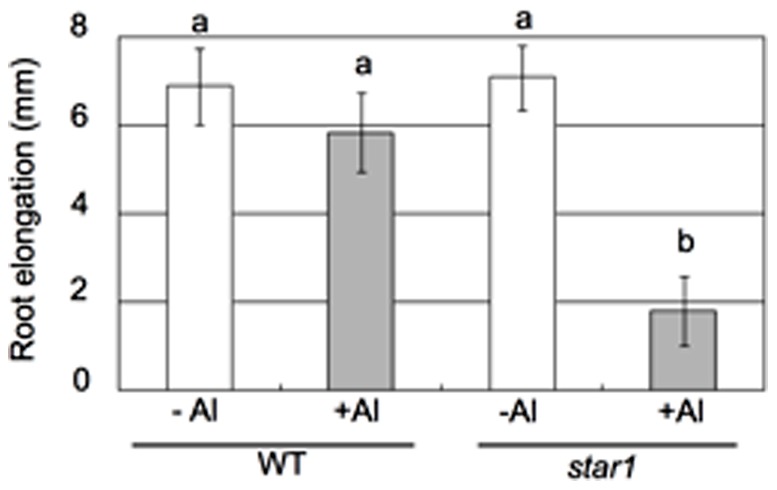
Al-induced inhibition of root elongation. Seedlings (6-d-old) of both wild-type rice (WT) and an Al-sensitive mutant (*star1*) were exposed to a 0.5 mM CaCl_2_ solution (pH 4.5) containing 0, 20 µM Al for 6 h. The root length was measured with a ruler before and after Al treatment. Error bars represent ± SD (*n* = 10). Different letters indicate significant differences at *P*<0.05 by Tukey’s Honestly Significantly Different test.

**Figure 2:**
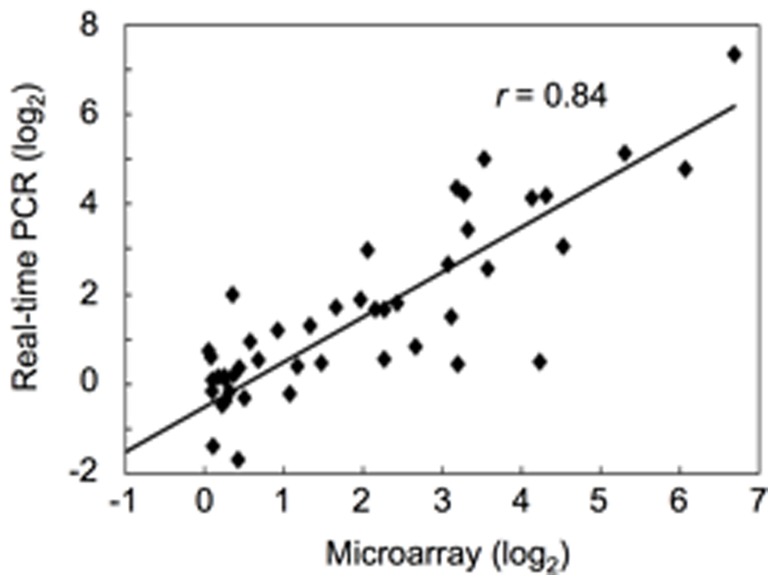
Correlation of gene expression ratio between microarray data and quantatitive RT-PCR data. Twelve genes randomly selected were subjected to quantitative real-time PCR analysis. OsHistone H3 was used as an internal standard. Microarray data (fold change of gene expression) were plotted against data (fold change of gene expression) from quantitative real-time PCR. Both x- and y-axes are shown in log^2^ scale. *r* indicates correlation coefficient.

### RNA Isolation, Microarray and Data Analysis

Root tips (0–1 cm) and basal region (1–2 cm) of the roots (20–30 plants per sample) were excised from the seedlings of both wild-type rice and *star1* mutant which had been exposed to 20 µM AlCl_3_ for 6 h and immediately frozen in liquid nitrogen. Total RNA was isolated using the RNeasy Plant Mini Kit (Qiagen, Germany). The RNA quality was assessed on agarose gels and with the Nanodrop ND-1000 (Thermo Fisher Scientific, USA). Microarray analysis was performed according to Agilent Oligo DNA Microarray Hybridization protocols using the Agilent 44 K Rice Oligo DNA Microarray RAP-DB (Agilent Technologies, USA; G2519F#15241) [15] with three biological replicates (Agilent Technologies, USA; G2519F#15241) [15]. The hybridized slides were scanned using a DNA microarray scanner (Agilent Technologies, USA). Signal intensities were extracted by Feature Extraction software (Agilent Technologies, USA). For statistical analysis, we excluded genes we excluded genes with low signal intensities less than 500 (sum of +Al and –Al signal intensity) in all treatments of the wild-type and *star1* mutant. This is based on expression level of known Al-tolerance genes (10–14). The average value (arithmetic mean) of fold change (the ratios of Cy3 and Cy5) and standard deviation (SD) of each probe were calculated using three biological replicates. Since the expression of known Al-tolerance genes is usually up-regulated by higher than three folds (10–14), we extracted genes up-regulated or down-regulated by Al more/less than three-fold in the wild-type and *star1* mutant. The data discussed in this publication have been deposited in NCBI's Gene Expression Omnibus and are accessible through GEO Series accession number GSE40964 (http://www.ncbi.nlm.nih.gov/geo/query/acc.cgi?acc=GSE40964).

**Figure 3:**
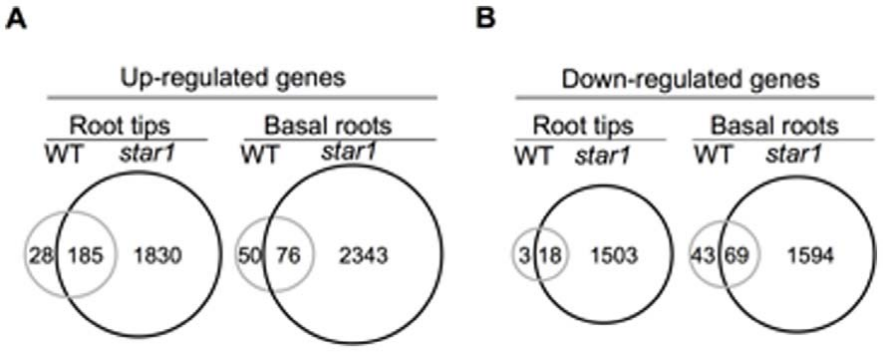
Genes up- and down-regulated by Al in the wild-type and *star1* mutant roots. Numbers of Al-responsive genes up-regulated (higher than 3-fold) (A) and down-regulated (lower than 3-fold) (B) are extracted. Wild-type (gray circle) and *star1* mutant (black circle) are shown in both the root tips and basal root region.

The gene functions were categorized based on databases including National Center of Biotechnology Information (NCBI) [16], the Rice Annotation Project Database (RAP-DB) build 5.0 [15] by the International Rice Genome Sequencing Project (IRGSP) [17], and the MSU Rice Genome Annotation Database [18]. The majority of Al-responsive transcripts were assigned to one of the following 12 categories by checking one by one using excel (Microsoft); (1) transport; (2) metabolism; (3) protein synthesis and processing; (4) signal transduction; (5) translation initiation or transcription factors; (6) abiotic or biotic stress response; (7) cell-wall, cell cycle, cell growth and cell cytoskeleton modification or metabolism; (8) DNA/RNA binding or metabolism; (9) phytohormone metabolism and response; (10) mitochondria or plastid; (11) other; (12) unknown molecular function.

**Table 1:** Functional classification of Al-responsive genes in the roots of the wild-type rice and *star1* mutant.

	Up-regulated^a^				Down-regulated^b^			
	Root tips		Basal roots		Root tips		Basal roots	
	WT (%)	*star1* (%)	WT (%)	*star1* (%)	WT (%)	*star1* (%)	WT (%)	*star1* (%)
Transport	19 (8.8)	125 (6.2)	10 (7.9)	113 (4.7)	1 (4.8)	102 (6.7)	4 (3.6)	135 (8.1)
Metabolism	31 (14.4)	251 (12.5)	18 (14.3)	340 (14.1)	4 (19.0)	119 (7.8)	19 (17.0)	204 (12.3)
Protein synthesis and processing	14 (6.5)	129 (6.4)	7 (5.6)	125 (5.2)	1 (4.8)	86 (5.7)	6 (5.4)	89 (5.4)
Signal transduction	6 (2.8)	147 (7.3)	2 (1.6)	140 (5.8)	0 (0)	97 (6.4)	9 (8.0)	98 (5.9)
Translation initiation or transcription factors	8 (3.7)	113 (5.6)	3 (2.4)	182 (7.5)	3 (14.3)	114 (7.5)	5 (4.5)	123 (7.4)
Abiotic or biotic stress response	39 (18.1)	276 (13.7)	13 (10.3)	269 (11.1)	3 (14.3)	126 (8.3)	25 (22.3)	133 (8.0)
Cell-wall, cell cycle, cell growthand cell cytoskeleton modificationor metabolism	14 (6.5)	67 (3.3)	13 (10.3)	133 (5.5)	1 (4.8)	137 (9.0)	9 (8.0)	94 (5.7)
DNA/RNA binding or metabolism	1 (0.5)	26 (1.3)	1 (0.8)	60 (2.5)	0 (0)	77 (5.1)	0 (0)	37 (2.2)
Phytohormone metabolism and response	2 (0.9)	21 (1.0)	3 (2.4)	26 (1.1)	0 (0)	22 (1.4)	1 (0.9)	17 (1.0)
Mitochondria or plastid	3 (1.4)	26 (1.3)	1 (0.8)	27 (1.1)	1 (4.8)	12 (0.8)	2 (1.8)	8 (0.5)
Other	0 (0.0)	19 (0.9)	2 (1.6)	31 (1.3)	0 (0)	12 (0.8)	1 (0.9)	14 (0.8)
Unknown molecular function protein	76 (35.2)	815 (40.4)	53 (42.1)	973 (40.2)	7 (33.3)	618 (40.6)	31(27.7)	711 (42.8)
Total	213	2015	126	2419	21	1521	112	1663

a Genes which expression was changed higher than 3-fold (fluorescence signal more than 500) in the root tips and the basal roots were categorized.

b Genes which expression was changed lower than 3-fold (fluorescence signal more than 500) in the root tips and the basal roots were categorized.

### Quantitative Real-time PCR

To validate microarray data, 12 genes were randomly selected for quantitative real-time PCR (qRT-PCR) (Table S1). Total RNA was prepared from the root tips and basal root regions of wild-type and *star1* using RNeasy Plant Mini Kit (Qiagen, Germany) and reversely transcribed using SuperSript™ II Reverse Transcriptase (Invitrogen, USA) and Oligo(dT) primers. The qRT-PCR was performed on an Eppendorf MasterCycler ep realplex real-time PCR (Eppendorf, Germany) using the specific primers described in Table S1.

**Figure 4:**
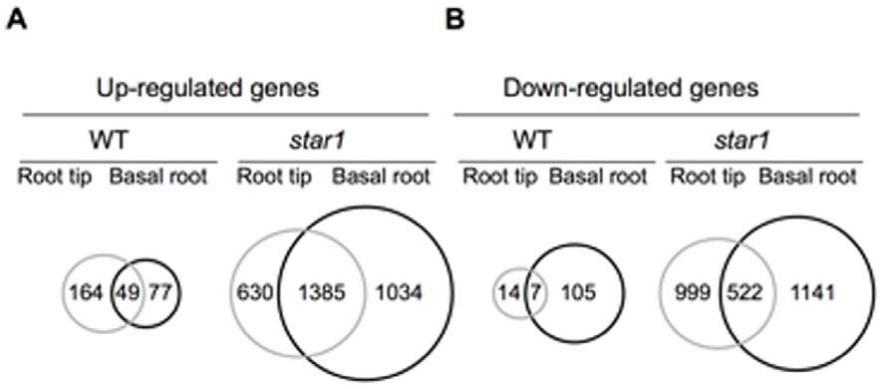
Genes up- and down-regulated by Al in the root tips and basal roots of the wild-type and *star1* mutant. Number of Al-responsive genes up-regulated (higher than 3-fold) (A) and down-regulated (lower than 3-fold) (B) are extracted. Root tips (gray circle) and basal root regions (black circle) was shown in the wild-type and *star1* mutant.

One-fifth dilutions of the cDNAs were used as a template for the qRT-PCR in a total volume of 20 µL as follows; 10 µL SYBR Premix Ex Taq™ Perfect Real Time (TaKaRa Biol Inc., Japan), 0.4 µL ROX Reference Dye, 0.8 µL primer mix (50∶50 mix of forward and reverse primers at 10 pmol µL^−1^ each), 6.8 µL distilled water and 2 µL template. The reaction conditions were: 30 s at 95°C followed by 40 cycles of 30 s at 95°C, 20 s at 60°C and 35 s at 72°C. The rice Histone H3 was used as an internal control. Relative expression levels were calculated by the comparative Ct method. Three independent biological replicates were made for each gene.

**Table 2:** Expression changes of ART1-regulated genes in the roots of wild-type rice and *star1* mutant.

			Root tips				Basal roots			
			WT		*star1*		WT		*star1*	
RAP ID^a^	Accession^b^	Annotation^c^	Fold change(+Al/−Al)^d^	±SD^e^	Fold change(+Al/−Al)	±SD	Fold change(+Al/−Al)	±SD	Fold change(+Al/−Al)	±SD
**Cell wall maintenance and Root elongation**										
Os01g0178300	AK062450	OsCDT3	7.43	1.58	24.67	3.28	11.72	3.85	9.82	2.66
Os01g0652100	AK069291	Protein of unknown function DUF231 domaincontaining protein	3.31	0.40	2.55	0.41	3.97	0.61	1.58	0.83
Os01g0860500	AK069860	Chitinase	10.32	4.51	12.09	2.34	3.67	0.84	7.41	4.20
Os03g0760800	AK121316	Gibberellin regulated protein family protein	4.91	1.29	21.94	6.13	7.54	2.07	9.13	5.14
Os04g0583500	AK062225	Expansin 4	5.28	1.23	1.36	0.42	1.01	0.19	1.47	0.84
Os09g0479900	CI269495	Peptidase S8 and S53, subtilisin, kexin, sedolisin domaincontaining protein	3.43	0.25	6.40	0.78	1.40	0.19	9.83	5.61
Os10g0524600	AK069238	Peptidase S8 and S53, subtilisin, kexin, sedolisin domaincontaining protein	2.26	0.15	3.46	0.76	10.59	2.88	25.71	11.89
**Membrane protein**										
Os01g0869200	AK073453	Mg^2+^ transporter/OsMGT1	4.43	0.77	2.12	0.42	3.24	0.59	1.78	0.48
Os02g0131800	AK102180	OsNramp4/OsNrat1	7.85	0.31	0.74	0.08	5.53	0.18	0.27	0.08
Os02g0755900	AK104985	UDP-glucuronosyl/UDP-glucosyltransferase family protein	5.91	0.28	23.35	1.40	1.21	0.29	44.24	6.83
Os03g0755100	AK066049	Tonoplast-localized half-size ATP binding cassette (ABC)transporter/OsALS1	3.43	0.15	4.21	0.18	2.57	0.09	2.63	0.74
Os05g0119000	AK069359	Bacterial-type ATP binding cassette (ABC) transporter/OsSTAR2	6.75	1.86	3.62	0.85	3.68	0.90	1.63	0.47
Os06g0695800	AK109450	Bacterial-type ATP binding cassette (ABC) transporter/OsSTAR1	4.39	0.18	4.60	0.33	3.88	0.19	2.41	0.15
Os09g0426800	AK060786	Gl1 protein	1.97	0.38	4.67	0.53	5.34	0.71	0.99	0.22
Os10g0206800	AK072077	Multidrug and toxic compound extrusion (MATE) family protein/OsFRDL2	5.76	0.21	3.44	0.30	5.89	0.44	1.58	0.29
Os10g0578800	AK065615	LrgB-like protein family protein	7.89	0.49	2.36	0.15	5.22	0.24	0.74	0.47
**Metabolism and Detxification**										
Os01g0716500	AK101454	SAM (and some other nucleotide) binding motif domaincontaining protein	1.90	0.10	42.65	4.84	1.99	0.30	13.98	12.53
Os02g0186800	NM_001052658	Cytochrome P450 family protein	12.48	4.64	5.23	0.59	5.80	0.90	1.99	0.22
Os02g0770800	AK102178	Nitrate reductase	8.63	1.34	10.99	0.45	30.19	4.29	59.40	7.64
Os12g0227400	CI560939	Allyl alcohol dehydrogenase	16.06	0.78	2.64	0.20	8.94	0.15	0.48	0.23
**Unknown**										
Os01g0731600	NM_001050684	Conserved hypothetical protein	18.18	4.39	23.14	5.08	1.68	0.31	14.15	13.58
Os01g0766300	NM_001050890	Conserved hypothetical protein	6.23	1.82	30.97	3.95	5.94	0.63	12.56	3.48
Os01g0919200	AK071325	Cell division protein FtsZ family protein	4.24	0.80	11.58	2.03	2.24	0.51	18.69	16.31
Os03g0126900	AK109217	Conserved hypothetical protein	7.32	0.67	4.11	0.24	7.84	0.47	2.11	0.08
Os03g0304100	AK111121	Hypothetical protein	10.73	4.09	34.63	11.22	4.04	0.49	0.78	0.57
Os04g0419100	AK107777	Hypothetical protein	16.41	0.44	5.38	0.39	1.09	0.23	7.96	5.25
Os04g0494900	AK073892	Protein of unknown function DUF642 family protein	15.05	0.86	3.64	0.74	2.98	0.16	99.56	31.86
Os07g0493100	AK068708	Non-protein coding transcript, uncharacterized transcript	26.08	11.10	7.18	0.87	13.41	4.30	6.97	2.12
Os07g0587300	CI285201	Hypothetical protein	6.62	1.76	208.44	71.70	6.36	1.10	104.49	46.28
Os11g0488100	CI197875	Hypothetical protein	4.50	0.16	7.00	0.56	2.02	0.12	1.96	0.13
Os11g0490100	AK108872	Uncharacterized plant-specific domain 01627 containing protein	5.04	0.72	1.76	0.25	9.75	1.10	21.53	10.50

a RAP-ID based The Rice Annotation Project (RAP) ID numbers.

b Accsesion based GenBank locus of the National Center of Biotechnology Information (NCBI).

c Annotation based on the Rice Annotation Project Database (RAP-DB) build 3.0 by the International Rice Genome Sequencing Project (IRGSP).

d Fold change, ratio of transcript abundance in Al treatement/transcript abundance in control (−Al) treatment.

e Standard deviation of the mean.

## Results and Discussion

Tolerance and toxicity of Al stress are a complicated phenomenon, involving many genes and a number of signaling pathways [19]. However, microarray technique has provided a useful tool for investigation of genome-wide changes in transcripts. So far, microarray analysis for Al response has been reported in Arabidopsis [20]–[22], maize [23], [24], *Medicago truncatula* [25], [26], and wheat [27]. Since the mechanisms for Al-tolerance differ with plant species, in the present study, we performed a microarray analysis with rice, a well-known Al-tolerant species, to understand genes involved in high Al-tolerance at genome-wide scale.

**Table 3:** Genes up- and down-regulated only in the root tips of wild-type rice.

Functional classificationf^a^/RAP ID^b^	Accession^c^	Annotation^d^	Fold change (+Al/−Al)^e^	±SD^f^
**Up-regulated**				
**ART1-regulated genes**				
Os12g0227400	CI560939	Allyl alcohol dehydrogenase	16.06	0.78
Os10g0578800	AK065615	LrgB-like protein family protein	7.89	0.49
Os02g0131800	AK102180	OsNramp4/OsNrat1	7.85	0.31
Os11g0490100	AK108872	Uncharacterized plant-specific domain 01627 containingprotein	5.04	0.72
Os04g0583500	AF247165	Expansin 4	5.00	1.49
Os01g0869200	AK073453	Mg^2+^ transporter/OsMGT1	4.43	0.77
**Other genes**				
**Transpot**				
Os05g0410900	AK119621	Nitrate transporter/OsNRT1	6.91	0.93
Os03g0667500	AY327039	Iron-regulated transporter 2/OsIRT2	3.16	0.28
**Metabolism**				
Os08g0468100	AK101662	Nitrate reductase [NADH] 1/OsNR	3.02	0.37
**Protein synthesis and processing**				
Os05g0360400	AK106046	Zn-finger, RING domain containing protein	5.30	0.93
Os04g0535200	AK060585	Peptidase aspartic family protein	3.25	0.33
**Translation initiation or transcription factors**				
Os07g0569100	AK120160	Remorin, C-terminal region domain containing protein	3.17	0.52
**Abiotic or biotic stress response**				
Os03g0804500	AF072694	Germin-like protein subfamily T member 1 precursor/OsGLP	4.68	0.49
Os07g0214900	NP_001059187	Chalcone synthase/OsCHS	4.36	0.24
Os04g0456200	NP_001052967	TMV induced protein 1–2	3.68	0.23
Os05g0495900	AB027431	Beta-1,3-glucanase precursor	3.68	0.38
Os01g0713200	AB027429	Beta-1,3-glucanase precursor	3.23	0.62
**Cell-wall, cell cycle, cell growth and cell cytoskeleton modification or metabolism**				
Os04g0664900	CI550916	Cell wall invertase	4.12	0.64
Os04g0683700	AK119512	4-coumarate-CoA ligase-like protein	3.38	0.16
Os07g0568700	AF466357	Floral organ regulator 1	3.35	1.09
**Hormone metabolism and response**				
Os03g0738600	AK073529	Lipoxygenase L-2	3.63	0.89
**Unknown molecular function protein**				
Os10g0137300	NP_001064130	Conserved hypothetical protein	7.55	0.91
Os03g0183200	AK106987	Conserved hypothetical protein	4.36	0.98
Os01g0915900	CI543502	(No Hit)	3.67	1.08
Os11g0211800	AK059202	Hypothetical protein	3.27	1.09
Os01g0824800	AK066200	Conserved hypothetical protein	3.13	0.62
Os01g0319200	NP_001042887	Plant protein of unknown function family protein	3.10	0.22
Os05g0410800	AK108312	Conserved hypothetical protein	2.99	0.08
**Down-regulated genes**				
**Translation initiation or transcription factors**				
Os07g0558100	Y11415	Myb protein (similar to ATMYB102)	0.27	0.02
Os03g0279700	AK111338	ZPT2-12	0.33	0.11
**Unknown molecular function protein**				
Os10g0391400	AK107854	ZIM domain containing protein. (simirlar to JAZ; JA signaling)	0.28	0.06

a Funcronal classification based on Table 1.

b RAP-ID based The Rice Annotation Project (RAP) ID numbers.

c Accsesion based GenBank locus of the National Center of Biotechnology Information (NCBI).

d Annotation based on the Rice Annotation Project Database (RAP-DB) build 3.0 by the International Rice Genome Sequencing Project (IRGSP).

e Fold change, ratio of transcript abundance in Al treatement/transcript abundance in control (−Al) treatment.

f Standard deviation of the mean.

Al-toxicity is characterized by inhibition of root elongation, which occurs within a few hours after exposure to Al [3]. Therefore, to exclude genes associated with Al-toxicity, we sampled the roots exposed to Al solution for 6 h for microarray analysis. Furthermore, to extract genes related to Al-tolerance, we compared the transcriptional profiling between the wild-type rice and an Al-sensitive mutant, *star1* [10]. Moreover, we selected a concentration of 20 µM for Al treatment. At this concentration, the root elongation of the wild-type rice was hardly inhibited, whereas that of the mutant was inhibited by 75% (Figure 1), which make possible to extract genes possibly associated with Al-tolerance.

### Verification of Microarray Results by Quantitative Real-time PCR

To validate the reliability of the microarray data, we randomly selected 12 genes from root tips and basal root regions for the quantitative real-time PCR (qRT-PCR) analysis. There was a good correlation (*r* = 0.84) between the microarray data and the qRT-PCR results (Figure 2). These results indicated that the microarray data could reflect the transcriptional changes caused by Al stress.

**Table 4:** Genes up- and down-regulated only in the basal roots of wild-type rice.

Functional classificationf^a^/RAP ID^b^	Accession^c^	Annotation^d^	Fold change(+Al/−Al)^e^	±SD^f^
**Up-regulated**				
**ART1-regulated genes**				
Os12g0227400	CI560939	Allyl alcohol dehydrogenase	8.94	0.15
Os03g0126900	AK109217	Conserved hypothetical protein	7.84	0.47
Os10g0206800	AK072077	Multidrug and toxic compound extrusion (MATE) family protein/OsFRDL2	5.89	0.44
Os02g0131800	AK102180	OsNramp4/OsNrat1	5.53	0.18
Os09g0426800	AK060786	Gl1 protein	5.34	0.71
Os10g0578800	AK065615	LrgB-like protein family protein	5.22	0.24
Os03g0304100	AK111121	Hypothetical protein	4.04	0.49
Os06g0695800	AK064089	Bacterial-type ATP binding cassette (ABC) transporter/OsSTAR1	3.81	0.19
Os05g0119000	AK069359	Bacterial-type ATP binding cassette (ABC) transporter/OsSTAR2	3.68	0.90
Os01g0869200	AK073453	Mg^2+^ transporter/OsMGT1	3.24	0.59
**Other genes**				
**Transport**				
Os06g0701700	AB061311	HKT-type transporter (Sodium ion transporter)	2.97	0.83
**Metabolism**				
Os08g0547300	AK072163	E-class P450, group I family protein	7.65	3.42
Os04g0405300	AK110700	Stem secoisolariciresinol dehydrogenase	4.04	1.05
Os06g0500700	CI431272	Cytochrome P450 family protein	3.48	1.21
Os02g0176900	NP_001046065	Aldose 1-epimerase family protein	3.47	0.67
Os05g0438600	AY035554	Fructose-1,6-bisphosphatase (FBPase)	3.45	0.16
Os11g0487600	NP_001067918	Cytochrome P450 family protein	3.17	0.09
Os05g0424300	AK120987	Cytochrome P450 family protein	3.07	0.44
**Protein synthesis and processing**				
Os12g0108500	AK122171	Cyclin-like F-box domain containing protein	10.16	0.26
Os04g0535200	AK060585	Peptidase aspartic family protein	3.10	0.35
**Translation initiation or transcription factors**				
Os01g0286100	AK102252	Basic helix-loop-helix dimerisation region bHLH domain containing protein	3.34	0.45
**Abiotic or biotic stress response**				
Os09g0361500	AK120689	Isochorismate synthase 1 (ICS1)	4.08	0.76
Os05g0223000	AK071661	Calmodulin-related protein 2, touch-induced	3.32	0.77
Os04g0635500	AK069933	Wound induced protein	3.05	0.81
**Cell-wall, cell cycle, cell growth and cell cytoskeleton modification or metabolism**				
Os04g0506800	AK070719	Glycosyl transferase, family 29 protein/OsGT	5.21	0.77
Os11g0444000	AK099588	UDP-glucosyltransferase BX8	3.50	0.37
Os02g0802200	AK107538	Glycoside hydrolase, family 79, N-terminal domain containing protein/OsGH	3.34	0.42
Os04g0477500	AK063950	Glycosyl transferase, family 17 protein/OsGT	3.15	0.30
Os03g0324700	AK121618	Exostosin-like family protein	3.03	0.25
**Hormone metabolism and response**				
Os04g0667400	AK119413	2OG-Fe(II) oxygenase domain containing protein	5.42	1.44
**Mitochondria or plastid**				
Os07g0469100	AK120365	Thylakoid membrane phosphoprotein 14 kDa	3.06	0.34
**Unknown molecular function protein**				
Os07g0269000	CI251879	(No Hit)	3.74	0.10
Os10g0473200	AK105229	Conserved hypothetical protein	5.05	0.76
Os05g0573800	CI142713	(No Hit)	4.49	0.56
Os04g0635400	CI037812	Conserved hypothetical protein	4.38	1.40
Os04g0603800	AK063616	Hypothetical protein	4.17	0.13
Os03g0183200	AK106987	Conserved hypothetical protein	4.10	0.75
Os12g0265400	CI096837	Hypothetical protein	4.05	0.23
Os09g0459900	AK063208	Cyclin-dependent kinase inhibitor family protein	3.92	1.21
Os09g0459500	AB118006	Hypothetical protein	3.87	0.49
Os03g0255500	AK061620	Phosphoenolpyruvate carboxykinase	3.69	0.36
Os01g0213500	CI426147	Conserved hypothetical protein	3.45	0.15
Os11g0259100	NP_001067644	Hypothetical protein	3.43	0.60
Os02g0600200	AK058978	IQ calmodulin-binding region domain containing protein	3.23	0.70
Os02g0327000	AK073631	C2 domain containing protein	3.15	0.28
Os06g0535200	AK109943	Zn-finger, RING domain containing protein	3.10	0.17
Os01g0854000	AK070440	Conserved hypothetical protein	3.10	0.72
Os04g0520700	AK065832	Protein of unknown function DUF584 family protein	3.08	0.81
Os03g0113900	AK119700	Protein of unknown function DUF584 family protein	2.98	0.19
Os04g0231800	AK068417	Protein of unknown function DUF1165 family protein	2.95	0.03
**Down-regulated**				
**Transport**				
Os04g0538900	CI558963	Glyoxalase/bleomycin resistance protein/dioxygenase domain containing protein	0.29	0.10
Os03g0817200	AK121940	Amino acid/polyamine transporter II family protein	0.31	0.03
Os03g0375900	AK107064	Amino acid/polyamine transporter I family protein	0.32	0.05
**Metabolism**				
Os06g0185500	C97337	Transferase family protein	0.05	0.02
Os06g0185300	–	Transferase family protein	0.15	0.04
Os12g0626400	AK063967	Squalene/phytoene synthase family protein	0.22	0.03
Os06g0549900	AK109673	FAD linked oxidase, N-terminal domain containing protein	0.23	0.07
Os06g0294600	AK058424	Cytochrome P450 family protein	0.24	0.01
Os11g0644800	CI019806	Tyrosine/nicotianamine aminotransferase family protein	0.25	0.04
Os07g0643400	AK061012	Esterase/lipase/thioesterase domain containing protein	0.31	0.02
**Protein synthesis and processing**				
Os01g0124100	AK062394	Proteinase inhibitor I12, Bowman-Birk family protein	0.13	0.01
Os10g0537800	AK061277	Peptidase A1, pepsin family protein	0.21	0.06
Os03g0318400	AK106440	Peptidase A1, pepsin family protein	0.21	0.07
**Signal transduction**				
Os07g0186200	NP_001059070	Protein kinase family protein	0.32	0.03
Os04g0618700	AK120799	Protein kinase domain containing protein	0.34	0.02
Os01g0699600	AK105196	Protein kinase domain containing protein	0.34	0.23
**Translation initiation or transcription factors**				
Os02g0624300	AK112056	MYB1 protein	0.11	0.09
Os11g0702400	AK105226	Zn-finger, C2H2 type domain containing protein	0.26	0.03
**Abiotic or biotic stress response**				
Os07g0129300	AF306651	Pathogenesis-related protein 1 precursor	0.07	0.02
Os06g0546500	AK073833	Peroxidase	0.16	0.03
Os05g0427400	CI551987	Phenylalanine ammonia-lyase	0.19	0.01
Os02g0627100	AK068993	Phenylalanine ammonia-lyase	0.22	0.04
Os09g0417800	AK067834	DNA-binding WRKY domain containing protein	0.24	0.07
Os09g0417600	AF467736	DNA-binding WRKY domain containing protein	0.26	0.05
Os10g0542900	AB016497	Chitinase	0.28	0.06
Os05g0135400	AK063587	Plant peroxidase family protein	0.29	0.06
Os05g0149400	AK061064	1-aminocyclopropane-1-carboxylate oxidase/OsACC	0.30	0.10
Os01g0687400	AB110201	Chitinase	0.31	0.04
Os11g0592000	AK121059	Barwin	0.33	0.04
Os01g0933900	AF309383	Glutathione transferase III(B)	0.35	0.02
**Cell-wall, cell cycle, cell growth and cell cytoskeleton modification or metabolism**				
Os02g0267200	CI377660	Alpha-expansin OsEXPA13	0.32	0.04
**Unknown molecular function protein**				
Os04g0368000	CI447876	(No Hit)	0.15	0.04
Os06g0587300	AK121885	Conserved hypothetical protein	0.05	0.03
Os06g0586000	AK063903	Conserved hypothetical protein	0.07	0.03
Os12g0437800	AK063833	CI2E	0.08	0.02
Os01g0796000	CI508923	(No Hit)	0.12	0.04
Os10g0391400	AK107854	ZIM domain containing protein	0.18	0.15
Os05g0368000	NP_001055341	Conserved hypothetical protein	0.20	0.03
Os06g0282000	CI563293	(No Hit)	0.21	0.03
Os06g0292400	CI409636	Embryogenesis transmembrane protein	0.27	0.02
Os02g0520100	AK072610	NUDIX hydrolase domain containing protein	0.28	0.01
Os03g0187800	AK105352	Protein of unknown function DUF250 domain containing protein	0.30	0.05
Os06g0155400	NP_001056850	Hypothetical protein	0.33	0.06

a RAP-ID based The Rice Annotation Project (RAP) ID numbers.

b Accsesion based GenBank locus of the National Center of Biotechnology Information (NCBI).

c Annotation based on the Rice Annotation Project Database (RAP-DB) build 3.0 by the International Rice Genome Sequencing Project (IRGSP).

d Fold change, ratio of transcript abundance in Al treatement/transcript abundance in control (−Al) treatment.

e Standard deviation of the mean.

### Overview of Al-induced Transcriptional Profiling

Agilent 44 K Rice Oligo DNA Microarray RAP-DB covers almost genes in rice genome [15]. In the root tips (0–1 cm) of wild-type rice, exposure to 20 µM AlCl_3_ for 6 h resulted in up-regulation of 213 genes and down-regulation of 21 genes (Figure 3A and 3B). By contrast, much more genes (2015 and 1521) were up- and down-regulated by the same treatment in the *star1* mutant (Figure 3A and 3B). In the basal root region (1–2 cm), 126 and 112 genes, respectively, was up- and down-regulated in the wild-type rice, whereas the numbers of up- and down-regulated genes were 2419 and 1663, respectively, in the *star1* mutant (Figure 3A and 3B).

Functional category analysis showed that 27.0–42.8% of the up- and down-regulated genes are assigned to unknown function (Table 1). Genes related to ‘Metabolism’ and ‘Abiotic or biotic stress response’ were mostly affected by Al stress in both the wild-type rice and *star1* roots (Table 1).

Since the root elongation was hardly inhibited in the wild-type rice, but severely inhibited in the *star1* mutant, three different groups for Al-responsive genes could be divided by comparing expression profiling between wild-type rice and *star1* mutant. Group 1 includes genes which are up- or down-regulated by Al only in the wild-type rice. These genes are probably involved in Al-tolerance. Twenty eight up-regulated and three down-regulated genes in the root tips, 50 up-regulated and 43 down-regulated genes in the basal root region, belong to this group (Figure 3A and 3B). Group 2 includes genes, which are up- or down-regulated by Al in both the wild-type rice and *star1* mutant. These genes are probably involved in Al-tolerance or -toxicity. There are 185 up-regulated and 18 down-regulated genes in this group in the root tip, 76 up-regulated and 69 down-regulated genes in the basal root region (Figure 3A and 3B, Table S2–S5). Genes in Group 3 are those up- or down-regulated only in the mutant. These genes are related to Al-toxicity and included 1830 up-regulated genes and 1503 down-regulated genes in the root tip, 2343 up-regulated and 1594 down-regulated genes in the basal root region (Figure 3A and 3B). Most genes in this group are also response to general stresses and found in microarray data of other plant species such as Arabidopsis [20], maize [23], [24] *M. truncatula* [25], [26] and wheat [27]. For example, the genes encoding a NADPH oxidase, peroxidase, oxalate oxidase, which are reactive oxygen species (ROS; O_2_^−^, H_2_O_2_) generators, were up-regulated (Table S2). Most types of abiotic stresses disrupt the metabolic balance of cells, resulting in enhanced production of ROS [28]. The accumulation of ROS such as ^1^O_2_, O_2_^−^, H_2_O_2_ and HO^•^, during abiotic stresses was considered to be a by-product of stress metabolism as well as an overall unwelcome by-product of aerobic metabolism [29]. These findings indicate that these genes are involved in arrest of plant root elongation in response to general stress.

### Spatial Profiling of Al-responsive Genes

Root tip has been considered as the target of Al-toxicity [3] based on root elongation inhibition, however, surprisingly, similar numbers of genes were up- and down-regulated by Al in the root tips and mature regions of both wild-type rice and *star1* mutant (Figure 4A and 4B). This result raises a question on whether the root tip is only the target of Al-toxicity. Among genes affected, 49 up-regulated and 7 down-regulated genes were the same between root tip and basal root region in the wild-type rice (Figure 4A and 4B), but most Al-responsive genes were different between the root tip and basal root region. This was the same in the *star1* mutant; 1385 up-regulated and 522 down-regulated genes were the same between the root tip and basal root region, whereas other genes showed root region-dependent (Figure 4A and 4B). These results suggest that basal root region is also a target of Al-toxicity in addition to the root tip. In *M. truncatula*, Al-induced gene expression is also found not to be restricted to the root tip [25]. In fact, some genes identified from rice were expressed in both the root tips and basal root region. For example, *OsFRDL4* was expressed in both the root tip and the mature root zones [11]. The expression of *OsSTAR1* and *OsSTAR2* was also induced in both regions [10]. These findings suggest that the basal root region is also involved in Al-tolerance and -toxicity.

### Transcriptional Profiling of ART1-regulated Genes in the Wild-type and the *star1* Mutant Roots

ART1-regulated Al-tolerance has been identified as a major mechanism responsible for high Al-tolerance in rice [7], [8]. We compared expression profiling of ART1-reguated downstream genes between wild-type rice and *star1* mutant. Among 31 downstream genes, 11 genes showed higher fold changes in the expression in the mutant than in the wild-type rice (Table 2), whereas 13 genes showed higher fold changes in the wild-type rice than in the mutant (Table 2). Seven genes showed similar fold changes in the expression between wild-type rice and mutant (Table 2). Six genes were only up-regulated in the wild-type rice, including genes encoding Expansin (Os04g0583500), Mg^2+^ transporter/OsMGT1 (Os01g0869200), OsNramp4/OsNrat1 (Os02g0131800), LrgB-like protein family protein (Os10g0578800), Allyl alcohol dehydrogenase (Os12g0227400) and uncharacterized plant-specific domain 01627 containing protein (Os11g0490100, Table 2). Among them, OsNrat1 (Al^3+^ transporter) and OsMGT1 (Mg^2+^ transporter) have been demonstrated to be involved in Al-tolerance [12], [14]. Although several ART1-regulated genes were also up-regulated in the mutant, the Al-tolerance was severally decreased, indicating that not a single gene, but multiple genes are required to function together for high Al-tolerance in rice.

### Novel Al-tolerance Mechanism in Rice

Among 28 genes only up-regulated in the root tips of the wild-type (Table 2, Table 3), 6 genes are ART1-regulated, indicating that there are other mechanisms for Al-tolerance except ART1-regulated pathway in rice. Seven genes out of 22 genes belong to unknown function group (Table 3), while other genes are related to transporter (nitrate transporter, iron-regulated transporter), metabolism (nitrate reductase), oxidative stress-responsive genes (germin-like protein), polysaccharide/cell wall metabolism (cell wall invertase, beta-1,3-glucanase precursor) and so on.

Genes encoding nitrate transporter1 (OsNRT1; Os05g0410900) and nitrate reductase (OsNR; Os08g0468100) were up-regulated by 6.9- and 3.0-fold in the root tips of wild-type rice (Table 3). OsNRT1 is a low-affinity transporter for nitrate uptake [30], while OsNR is responsible for the reduction of nitrate to nitrite [31]. Rice takes up nitrogen mainly in the form of ammonium, therefore, it is unlikely that up-regulation of *OsNRT1* and *OsNR* is for enhancing nitrogen uptake. One possibility is that the up-regulation is associated with nitric oxide (NO) production. Nitric oxide is produced from nitrite and a key signal molecule involved in many physiological processes in plants [32]. In fact, addition of exogenous NO enhanced Al-tolerance in rice roots by decreasing the contents of pectin and hemicellulose, increasing the degree of methylation of pectin, and decreasing Al accumulation in root cell walls [32], supporting that up-regulation of OsNRT1 and OsNR is required for Al-tolerance in rice.

Gene encoding iron-regulated transporter 2 (OsIRT2; Os03g0667500) was up-regulated by 3.2-fold (Table 3). Fe uptake is proposed to be mediated through OsIRT1 and OsIRT2 [33]. Interestingly, only IRT2, but not OsIRT1 was up-regulated by Al. Furthermore, this up-regulation seems to be distinct in rice since its homolog is not induced by Al in Arabidopsis, maize, *M. truncatula*, and wheat roots [20]–[27]. Al inhibits Fe uptake [34], therefore up-regulation of OsIRT2 is necessary for increasing Fe uptake.

Genes related with secondary metabolism were also up-regulated by Al. Chalcone synthase (CHS, EC 2.3.1.74) is a key enzyme of the flavonoid/isoflavonoid biosynthesis pathway. A gene encoding this enzyme was up-regulated by 4.4-fold (Table 3). CHS is quite commonly induced in different plant species under different forms of stress like UV, wounding, herbivory and microbial pathogens, resulting in the production of compounds that have e.g. antimicrobial activity (phytoalexins), insecticidal activity, and antioxidant activity or quench UV light directly or indirectly [35]. CHS expression causes accumulation of flavonoid and isoflavonoid. On the other hand, 4-Coumarate:CoA ligase has a pivotal role in the biosynthesis of plant secondary compounds at the divergence point from general phenylpropanoid metabolism to several major branch pathways [36]. Al is known to induce peroxidation and ROS formation in rice roots [37], [38]. Increased secondary metabolites such as flavonoids may increase anti-oxidative capacity, subsequently alleviating Al-toxicity. In line with this aspect, a gene encoding germin-like protein (OsGLP; Os03g0804500) was also up-regulated (Table 3). Germin-like proteins (GLPs) constitute a diverse family of ubiquitous plant glycoproteins [39]. Many GLPs have manganese-containing superoxide dismutase (SOD) activity [40], [41]. The SOD activities catalyze the dismutation of superoxide into oxygen and hydrogen peroxide. In this study, OsSOD was not up-regulated in rice roots after short exposure to Al stress (Table 3, Table 4, Table S2, S3), indicating OsSOD might not function in rice root after short exposure to Al stress. Thus, OsGLP might function as SOD. Furthermore, the H_2_O_2_ produced by OsGLPs is detoxicated by peroxiredoxin (PrxR) and thioredoxin (Trx) because they are only up-regulated antioxidant genes in rice root tips (Table S2). These results suggested that OsGLP, OsPrxR and OsTrx function as major ROS-scavenging enzymes in the rice roots after short exposure to Al stress.

Among genes up-regulated by Al only in the basal region of wild-type rice, some are related to polysaccharide/cell wall metabolism, including genes encoding glycoside hydrolase (GH; Os02g0802200) and glycosyl transferases (GTs; Os04g0506800, Os04g0477500) (Table 4). Glycoside hydrolases (GHs) catalyze the hydrolysis of the glycosidic linkage to release smaller sugars [42]. Glycosyl transferases (GTs) catalyze the transfer of sugar moieties from activated donor molecules to specific acceptor molecules, thereby forming glycosidic bonds [43]. Al causes the thickening and rigidification of cell walls [44]. Increased expression of OsGH and OsGTs may contribute to the cell wall synthesis, hence alleviating the Al-induced inhibition of longitudinal cell expansion.

Gene encoding 1-aminocyclopropane-1-carboxylate oxidase (OsACC; Os05g0149400) was down-regulated in the basal root (Table 4). OsACC is related to biosynthesis of ethylene. Ethylene production is associated with inhibition of root elongation in *Lotus japonicus* and *M. truncatula* [45]. Down-regulation of OsACC may prevent further inhibition of root growth caused by Al. The association between other genes and Al-tolerance remain to be examined in future.

As a conclusion, our comparative genome-wide transcriptional analysis reveals that there are other mechanisms for Al-tolerance except for ART1-regulated one in rice including those related to nitrogen assimilation, secondary metabolite synthesis, cell-wall synthesis and ethylene synthesis. Although the exact roles of these putative tolerance genes remain to be examined, our data provide a platform for further work on Al-tolerance in rice.

## Supporting Information

Table S1 **Primer sequences used for quantitative real-time PCR.**
(XLS)

Table S2 **Genes up-regulated in the root tips of both the wild-type and** ***star1*** **mutant.**
(XLS)

Table S3 **Genes up-regulated in the basal root regions of both the wild-type and** ***star1*** **mutant.**
(XLS)

Table S4 **Genes down-regulated in the root tips of both the wild-type and** ***star1*** **mutant.**
(XLS)

Table S5 **Genes down-regulated in the basal roots of both the wild-type and** ***star1*** **mutant.**
(XLS)
